# Practice of distributed machine learning in clinical modeling for chronic obstructive pulmonary disease

**DOI:** 10.1016/j.heliyon.2024.e33566

**Published:** 2024-06-28

**Authors:** Junfeng Peng, Xujiang Liu, Ziwei Cai, Yuanpei Huang, Jiayi Lin, Mi Zhou, Zhenpei Xiao, Huifang Lai, Zhihao Cao, Hui Peng, Jihong Wang, Jun Xu

**Affiliations:** a Department of Computer Science and Engineering, Guangdong University of Education, Guangzhou 510303, China; b Third Affiliated Hospital of Sun Yat-Sen University, Guangzhou 510640, China

**Keywords:** Chronic obstructive pulmonary disease, Federated learning, Privacy protection, Real-world data

## Abstract

**Background:**

The high prevalence, morbidity and mortality, and disease heterogeneity of chronic obstructive pulmonary disease (COPD) result in the scattered data derived from patient visits in different medical units. The huge cost of integrating the scattered data for analysis and modeling, as well as the legal demand for patient privacy protection lead to the emergence of data island.

**Objectives:**

On the premise of protecting patient privacy, integrating scattered data of patients from different medical units for high-quality modeling is beneficial to promoting the development of digital health. Based on this, we develop a distributed COPD disease diagnosis system termed COPD average federated learning (COPD_AVG_FL) using FedAvg.

**Methods:**

First, to build the COPD_AVG_FL, the clinical data of COPD patients from the real world is collected and the data pre-processing is performed to clean the incorrect data, outlier samples and missing values. Then, a classical federated learning architecture is designed as COPD_AVG_FL. Finally, to evaluate the established COPD_AVG_FL system, we develop Centralized Machine Learning (CML).

**Conclusions:**

Our results suggest that, with the assistance of COPD_AVG_FL, the absolute improvement rates are 13.4% (accuracy), 13.3% (precision), 12.8% (recall), 13.1% (F1-Score) and 12.9% (AUC) on the test data, respectively. The decoupling between model training and raw training data protects the patients' privacy, and helps to securely integrate more COPD data from different medical units to generate a more comprehensive model COPD_AVG_FL. This approach promotes the landing of wise information technology of medicine for COPD in the real clinical world. Code for our model will be made available at https://github.com/Cczhh/COPD_AVG_FL/tree/master.

## 1 Introduction

Chronic obstructive pulmonary disease (COPD) as a common lung disease is characterized by shortness of breath, cough, lacking in strength, and repeated aggravation, which leads to a significant reduction in the quality of patient's life [1]. Tobacco smoking, pollution of indoor and outdoor, occupational dust (fume) exposure, immunity drop and genetics are the main causes of COPD [2], [3], [4]. COPD is the third cause of death worldwide, and more than 3 million people die of COPD every year, accounting for an estimated 6% of global deaths [5], [6]. COPD as a gender neutral disease is prevalent in lower and middle regions, and the average COPD age is 35-40 [7], [8]. COPD also causes a huge economic burden to the country. The economic cost of United States in 2020 reaches $49 billion [9]. While this cost of United Kingdom is estimated to £3.8 billion yearly [10].

Clinically, COPD is classified into two categories: the stable phase and the acute exacerbation phase. COPD patients in stable stage can use inhaled anticholinergic drugs alone and cooperate with appropriate outdoor activities to enhance their physique. While acute exacerbation of chronic obstructive pulmonary disease (AECOPD) causes a sudden worsening of respiratory symptoms such as breathlessness, persistent bad cough, excessive phlegm and the increased wheeze [11]. In those who suffer AECOPD period, the timely and reliable medical interventions like oxygen therapy, antibiotics and corticosteroids are needed [3], [12].

Global Initiative for Chronic Obstructive Lung Disease (GOLD 2021) categorizes the severity of COPD into: GOLD 1 (Mild), GOLD 2 (Moderate), GOLD 3 (Severe), and GOLD 4 (Very severe) based on the pulmonary function score [3], [13]. Patients with GOLD 3 or GOLD 4 require more timely medical intervention. GOLD 2022 has changed due to the prevalence of corona virus disease 2019 (COVID-19), the ABCD evaluation tool is still being used to estimate the symptom burden of COPD [14]. According to the latest guidelines, GOLD 2023, has introduced significant changes, with one of the key revisions being the transformation of the previous ABCD classification into ABE grouping. Individuals at high risk of acute exacerbations are no longer categorized based on the severity of symptoms but rather unified under the designation ‘high-risk acute exacerbation group’ [15]. This entails the merger of the former C and D groups into a single E group. GOLD is a significant reference in COPD treatment, but incompletely guides COPD therapy. At present, COPD cannot be completely cured, but can be prevented in advance and alleviated by treatment [16]. The development of the disease can be controlled by smoking cessation, cold prevention, inhaling ICS/LABA medications and, Long-term oxygen therapy (LTOT), after suffering from it [17], [18]. Several works have indicated that although COPD is incurable, early intervention in patients with COPD reduces the mortality of the disease.

Artificial intelligence (AI) has greatly contributed to the advancement of medical technology and digital health recently, providing a way to reduce mortality for COPD. COPD data generated during the treatment is beneficial to the follow-up analysis of clinical medication and treatment effect. In recent years, the rapid development of AI has made it possible to realize disease-specific data mining. Kronborg et al. proposed a two-layer probabilistic model to improve the telemedicine patient classification accuracy. In the two-layer probabilistic model, the features selected were based on oximetry, pulse rate, and blood pressure. The result may be more generalizable if more training data is provided [19]. Mahmudah et al. used data balancing and selected 825 features to model and predict the presence of COPD. The experiment showed high AUC score was achieved by elastic net regression and multiclass logistic regression with AUC of 89% and 90% [20]. Wang et al. employed the support vector machine (SVM) to identify AECOPD patients with 0.80 sensitivity and 0.83 specificity. The work proved that expanding the data source center may enhance the generalization ability of the method [21]. Srivastava et al. utilized the convolutional neural network (CNN) to assist medical professionals in detecting COPD using respiratory sounds and obtained 0.93 classification accuracy. This study selected five features: mel-frequency cepstral coefficients (MFCC), melspectrogram (mel-spectrogram), chromagram calculated from the waveform/power spectrogram (chroma_stft), constant-Q chromagram (chroma_cqt) and chroma_cens (chroma energy normalized variant (CENS)). If the diversity of the data is expanded, this can contribute to improving the model generalization [22]. Huang et al. developed a multiple locally weighted linear regression (LWLR) model for the occurrence of AECOPD and achieved the minimum prediction error of 9.03%. The study selected the local sulfur dioxide (SO2), nitrogen dioxide (NO2), carbon monoxide (CO) and particulate matter 2.5 (PM2.5) concentrations as the features. This study may improve by incorporating multiple factors on COPD incidence [23]. Hussain et al. developed 24 feature voting ensemble classifiers to identify the severity of COPD. The experimental result showed that the accuracy rate was 91.08%, and the AUC score was 96.87%. Collecting more data would be beneficial for improving model performance [24]. Ali et al. implemented a model based on soft voting score to predict the severity of idiopathic pulmonary fibrosis (IPF) disease, and finally obtained an accuracy of 0.71 and F1-Score of 0.66. The feature selected in this study was gender, height, weight, body mass index (BMI), etc. The results can be improved if more data is collected [25]. Siddiqui et al. used long short-term memory (LSTM), CNN, etc., to predict COPD with the vital signs measured by the non-invasive method of impulse-radio ultra-wide band (IR-UWB) radar. Age, gender, smoking situation, respiration per minute (RPM), etc., were selected. The experimental result showed that LSTM (93% accuracy) was superior to all employed models used. Nevertheless, there is potential for the model to improve as the number of trained samples increases [26]. ALTAN et al. employed a deep extreme learning machines classifier (deep ELM) with a lower-upper triangulation (LuELM) autoencoder to differentiate between five COPD severities with a classification accuracy of 94.31% based on 12-channel lung sounds [27]. Centralized Machine Learning (CML) is applied in scenarios where data from multiple sources can be centralized into a single node. In contrast, decentralized machine learning (DML) refers to the distribution of data among multiple participants or nodes, rather than being centralized in a single node or authoritative entity [28]. The advantages of DML include enhancing user data privacy protection and data exclusivity.

We are concerned with the exploration of the different methods above for predicting COPD, and it is worth noting that the generalization ability of the model is improved if the amount of data collected increases. A common flaw of these works is that the data used to train the AI model comes from their own independent medical units, which may lead to model prediction deviation. Considering the characteristics of high heterogeneity, high morbidity and high mortality of COPD, in the real world, patients autonomously choose their healthcare facilities based on factors such as their place of residence, medical condition, and transportation convenience. Consequently, data pertaining to COPD patients is scattered across various medical institutions. It is common that COPD data scatter and store separately in different medical units that vary in size and level. A simple way to mitigate the model prediction deviation problem is to send these data distributed in different medical units to a single node for analysis and modeling. It must be admitted that integrating data from multiple medical units is able to build a model with better performance, but the risk of patient privacy disclosure is greatly increased.

With the improvement of privacy awareness, people pay more attention to risk of data leakage. COPD data, as a subset of medical data, also faces data leakage risk. To protect the patient privacy, a number of laws have been enacted in healthcare. For instance, European Union enactes General Data Protection Regulation (GDPR) [29], United States promulgates the Health Insurance Portability and Accountability Act (HIPAA) [30], and the criminal law stipulates that divulging over 50 pieces of information is convicted in China. Consequently, the practice of centralized training models by copying patient data directly to the single node can lead to the leakage of private data and the risk of breaking the law. Thus, the tradeoff between high quality model building and patient privacy protection is key to the implementation of wise information technology of medicine (WITMED) and digital health for COPD in real world research. WITMED is short for wise information technology of medicine, referring to smart healthcare [31].

An approach that has been successful in the privacy protection in the distributed scenario is the use of federated learning (FL) [32]. FL is a distributed machine learning framework in which multiple clients are trained locally under the coordination of a central server. At present, FL is mainly applied in blockchain and fake news identification. Shen et al. proposed a distributed FL to predict the rail transit passenger flow based on blockchain [33]. Wang et al. employed the FL and zero-knowledge proof to explore the blockchain privacy protection mechanism in financial transaction services [34]. Ouyang et al. applied the FL based on the self-attention-based pretrained model BERT and deep convolutional neural network to detect fake news of COVID-19 [35]. Fu et al. developed a vertical federated boosting decision tree (GBDT) system VF^2^ for cross enterprise machine learning and gained 12.8-18.9 times faster than the existing vertical federation in terms of computational efficiency [36]. Boualouache et al. implemented a detection scheme based on FL to compute passive mobile attackers in the 5G vehicular edge, under the protection of vehicle privacy [37]. In order to detect and mitigate poisoning attacks in FL, zhao et al. presented a poisoning defense mechanism using server side generative adversarial network (GAN) [38]. Balta et al. constructed analytical frameworks applicable to government scenarios, demonstrating that accountability of the FL process is critical to overcome legislative and jurisdictional restrictions [39].

**Figure 1:**
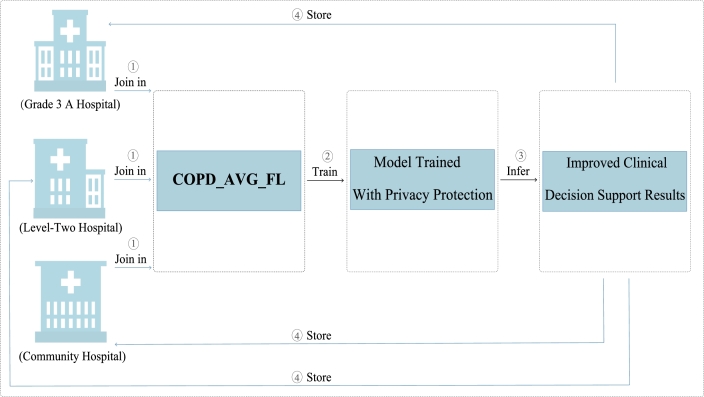
Process of joint modeling with multiple medical units based on COPD_AVG_FL (Hospital grading criteria is defined by China National Health Commission).

To sum up, FL is an appropriate approach to overcome the hindrance of data island to digital health. Considering the particularity of COPD data, building high-quality models using the scattered data under the premise of protecting patients' privacy is of great significance. Motivated by that, this paper design a FL architecture aiming at protecting patients' privacy, termed as COPD average federated learning (COPD_AVG_FL). COPD_AVG_FL system breaks the silos among medical units and achieves data integration. Fig. 1 represents the process of joint modeling with multiple medical units based on COPD_AVG_FL. Firstly, Grade 3 A hospital, Level-Two hospital and Community hospital are united through COPD_AVG_FL system. Then, the machine learning model is trained to infer the clinical diagnosis for COPD using the developed COPD_AVG_FL without the privacy exposure. Finally, the clinical diagnosis are stored to the hospitals joined.

For the contribution, our work has two innovative points:

1) The proposed COPD_AVG_FL system as a distributed learning framework with privacy-preserving capabilities tailors for COPD, in contrast to CML.
2) The system achieves an effective COPD classification performance by training a distributed architecture, to learn latent knowledge from COPD data originating from various medical units, thereby breaking down data island.

The rest of this paper is presented below. The dataset, principle and algorithm of COPD_AVG_FL system are depicted in section 2. COPD_AVG_FL is evaluated in Sec. 3. The discussion is carried out in Sec. 4. Finally, we conclude our work.

## 2 Methodology

**Figure 2:**
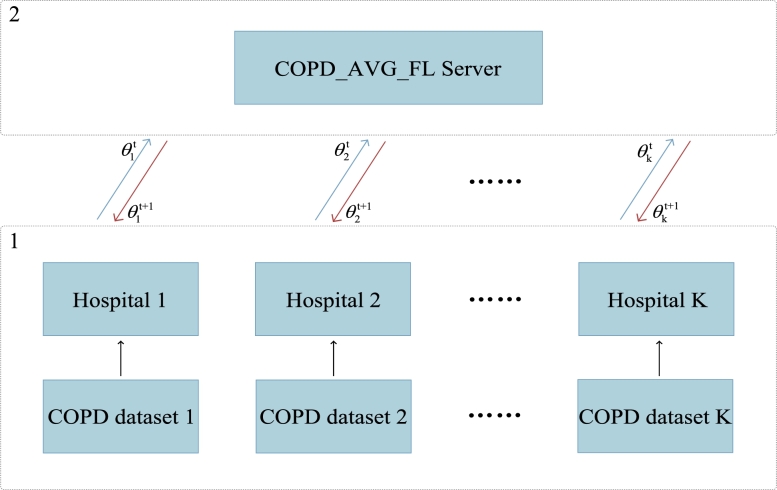
The COPD_AVG_FL framework.

To further the digital health of COPD, we introduce the FL approach proposed by McMahan et al. in 2016 to build COPD_AVG_FL [32]. A diagram of the developed COPD_AVG_FL can be found in Fig. 2. We can find that two major components of the developed COPD_AVG_FL: clients (hospitals) and COPD_AVG_FL server. The interaction between two main components is depicted as below: the client transmits the locally trained parameters to FL server after model parameters initialization, and then FL server returns the aggregation results of parameters to each client (hospital). Unlike the case of centralized training, COPD_AVG_FL achieves the high quality model construction with patient privacy protection only by transferring model parameters instead of patient related data. The dataset, principle and algorithm of the system are described in detail as follows.

### 2.1 Dataset

**Figure 3:**
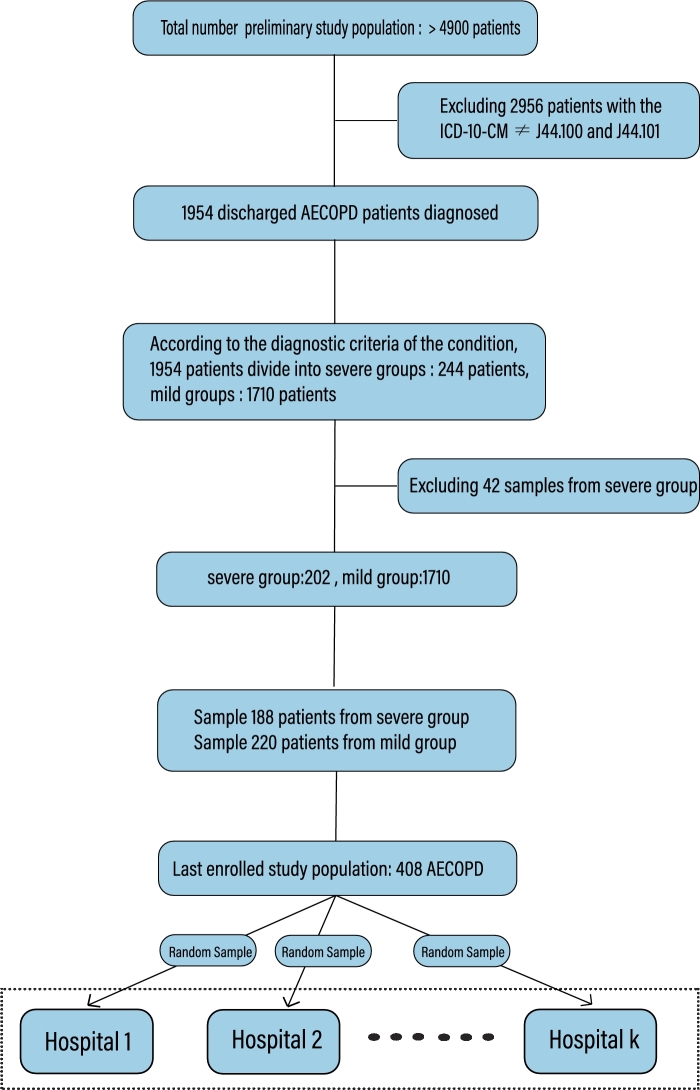
Flowchart of participants.

To evaluate the effectiveness of the proposed method, we obtain data from the Third Affiliated Hospital of Sun Yat-sen University (TAHSYU) for more than 4900 COPD patients with 248 clinical features from 2011 to 2018, delete the features with a hollow value greater than 10% in the medical records of the initial study population, and then use 40 features for modeling according to the suggestions of the clinician. 1954 AECOPD patients are screened from the initial study population using the international classification of diseases, Tenth Revision, clinical modification (ICD-10-CM) codes J44.100 and J44.101. This real world dataset from a reparable medical unit serves as a valuable resource for assessing the effectiveness of our approach. COPD is a common disease scattered in various medical units. Data collection is approved by the institutional review board (IRB) (scheme [2019]-02-334-01). Different from clinical trial data with strict process control, real-world data are inevitably incomplete, noisy and inconsistent. The flowchart of participants is shown in Fig. 3. According to the hospitalization situation, the severe and mild AECOPD patients are distinguished from 1954 AECOPD patients, including 244 severe patients and 1710 mild patients. 42 AECOPD patients without complete clinical data are excluded. 188 samples from 202 severe patients and 220 samples from 1710 mild patients are selected for modeling, and the sampling ratio is close to 1:1. Finally, 408 data records are determined as the input of the system. The input of the framework covers 345 male and 63 female patients. For statistical convenience, not all clinical features are included in Table 1. We then apply a simple random sampling, a common sampling method for simulating multiple data sources, to evaluate the performance of the proposed method in the auxiliary diagnosis of COPD.

**Table 1:** Description of experimental data (values are expressed as 95% CI of the mean).

	Thinning feature		Composition of patients	
			Mild group	Severe group
Number of cases			220(53.9%)	188(46.1%)

Sex	Male		188(85.5%)	157(83.5%)
	Female		32(14.5%)	31(16.5%)

Age (CI=95%)			(77.6, 80.0)	(80.2, 82.8)

Number of hospitalizations (CI=95%)			(3.3, 3.9)	(5.5, 7.5)

Blood pressure (CI=95%)	Systolic pressure		(130.6, 135.6)	(128.1, 135.1)
	Diastolic pressure		(74.2, 77.4)	(72.5, 76.3)

Temperature (CI=95%)			(36.7, 36.9)	(36.6, 36.8)

Pulse (CI=95%)			(90.5, 94.5)	(96.6, 101.4)

Respiratory rate (CI=95%)			(21.3, 22.1)	(23.8, 25.6)

### 2.2 Principle of COPD_AVG_FL

COPD_AVG_FL is a distributed learning system to construct a high-quality auxiliary diagnosis for COPD based on the typical federated averaging (FedAvg) [40]. The clients (hospitals) and FL server are the two main parts of the system. Each client (hospital) of COPD_AVG_FL locally computes the update of parameters with the gradient descent method using its local COPD dataset using  (1), then the server of COPD_AVG_FL aggregates the parameters of each client and returns the weighted average of the parameters to clients (hospitals) via  (2).

 is expressed by Equation (1):

(1) θ=θ−η▿L(θ)

where θ=<W,b> indicates iteration of various hospitals including weight and bias. θ=θ−η▿L(θ) denotes the local model updating with gradient descent, and *η* represents learning rate. Given that COPD diagnosis is a classification problem, we choose the cross-entropy function to calculate the loss. The loss function is expressed as $L=\frac{1}{N}\sum_{j}L_{j}=\frac{1}{N}\sum_{j}-[y_{j}\cdot \log(p_{j})+(1-y_{j})\cdot \log(1-p_{j})]$. $y_{j}$ represents the severity level of the j-th patient labeled by the physicians, 0 for the mild group and 1 for the severe group. $p_{j}$ indicates the probability that patient *j* is predicted to be the severe group.  is depicted by Equation (2):

(2) $$\theta^{r+1}=\frac{1}{K}\sum_{k=1}^{K}\theta_{k}^{r}$$

where *K* denotes the number of hospitals participating in FL, and *r* represents communication round. To calculate the global update quickly, the arithmetic average method is utilized to aggregate the uploaded parameters from each hospital.

### 2.3 Algorithm of COPD_AVG_FL

After principle description, the design of the COPD_AVG_FL algorithm is as follows. The inputs of COPD_AVG_FL include the local data from *K* hospital *D*, the number of local epoch *E*, the local minibatch size *B*, the learning rate *η*, and the number of communication rounds *r*. The output is *RES*, the predication results from multiple hospitals. The COPD_AVG_FL algorithm consists of two processes, first, : model parameter update process of client using gradient descent shown by Equation (1), then : global parameters update process aggregated by the COPD_AVG_FL server shown by Equation (2). The procedure is given in Algorithm 1. Step 1 to step 6: parameter updates for client models. Step 9: initialize three parameters, the current model $\theta_{cur}$, the next model $\theta_{next}$, and the predication results from multiple hospitals *RES*. Step 10 to step 19: for each communication round *r*, first for each client *k* call  function to update the local model $\theta_{k}^{r}$, then use averaging method to aggregate the updated parameters $\theta^{r+1}$, next for each client *k* calculate the prediction results $res_{k}$, next send the weighted average of the parameters to the clients. Step 20: finally return the predication results *RES* from multiple hospitals.  (The first line of Algorithm 1 function) and  (The eighth line of Algorithm 1 function). The hyperparameters of  encompass *r* (Communication rounds) and *K* (Number of clients which are parallel during training), while the hyperparameters of  comprise *E* (Epoch), and *I* (Iterations) denotes iterations of an epoch of model training. The computational complexity, expressed in big O notation, approximately O(r⁎E⁎I).

**Algorithm 1:**
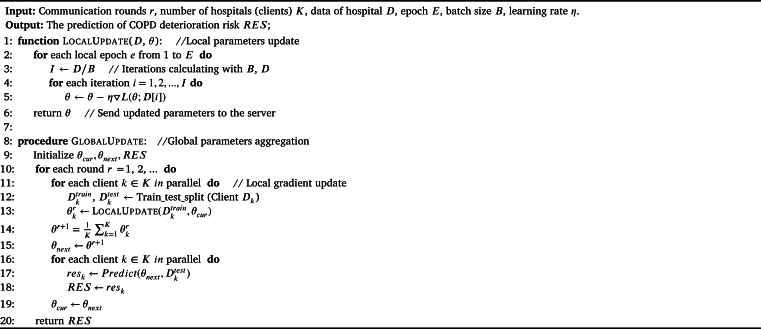
COPD_AVG_FL.

## 3 Evaluation

We report the evaluations aiming to validate the effectiveness of the developed COPD_AVG_FL system over the AVG_FL for the case of building a high quality model under the premise of protecting patient privacy [41]. In what follows, we present the metrics and the results of COPD_AVG_FL.

### 3.1 Metrics

We consider CML as the baseline in our evaluation. CML, dubbed baseline, uses isolated data from each hospital to train model and perform prediction tasks. For fair comparison with the implemented COPD_AVG_FL system, except that AVG_FL employment, the settings of COPD_AVG_FL and CML are the same in terms of model input, split ratio, backbone network, etc. In terms of specific evaluation indicators, the precision, recall, accuracy, F1-Score and receiver operating characteristic curve (ROC) [42] are adopted. Accuracy is used to evaluate the classification accuracy of the model. Similarly, recall, precision, F1-Score and ROC are used to evaluate other key capabilities of the model. These metrics provide a balanced evaluation embraced by data scientists. The metrics can be summarized as follows:

(3) $$Accuracy=\frac{TP+TN}{TP+TN+FP+FN}$$

(4) $$Precision=\frac{TP}{TP+FP}$$

(5) $$Recall=\frac{TP}{TP+FN}$$

(6) $$F1-Score=\frac{2\cdot Precision\cdot Recall}{Precision+Recall}$$

where TP, TN, FN and FP represent True Positives, True Negative, False Negatives and False Positives, respectively.

### 3.2 Results

To verify the effectiveness of the proposed architecture, we collect the COPD data from the real world, and declare the indispensable hyperparameters in Table 2. For the scientific aspects of hyperparameter selection of COPD_AVG_FL: in the FL architecture, the clients participating in this architecture are variable, so *K* is used to record the number of clients. In general, central server requires continuous communication with clients to make the model trained converge, so *r* is used to record communication rounds. For the model training of client, the client downloads the initialization parameters of the model from the central server. When it trains the model using its own data, *B* (Batch size), *E* (Epoch), *η* (Learning rate) and *T* (Train_test_split ratio) are the necessary hyperparameters. *K*, *r*, *E*, *B* are positive integers, while *η* and *T* are positive decimals. For simulation experiment simplicity and convenience, the range of client number is 2-5 and the default value is 3 experimentally. The range of communication rounds is 1-1000 and the default value is 350. The range of epoch is 3-10 and the default is 5. The range of batch size is 5-15 and the default is 10. The range of learning rate is [0.01, 0.001, 0.0001, 0.00001, 0.000001] and the default is 0.001. The range of train_test_split ratio is [5:5, 6:4, 7:3, 8:2, 9:1] and the default is 7:3.

**Table 2:** The hyperparameters of COPD_AVG_FL.

Hyperparameters	Description	Values
*K*	Number of clients	3
*r*	Communication rounds	350
*E*	Epoch	5
*B*	Batch size	10
*η*	Learning rate	0.001
*T*	Train_test_split ratio	7:3

Considering that the experimental data is structured, fully connected network (FCN), a simple and efficient neural network, is employed as the backbone of COPD_AVG_FL.

Accuracy represents the proximity between the predicted result and the ground truth. Precision refers to the probability of real cases in the samples with positive prediction. Recall indicates the proportion of positive cases in the original sample that are correctly predicted, which is a measure of the sample detection coverage of the classifier. Table 3 shows the performance evaluation comparison between the baseline CML and COPD_AVG_FL based model on accuracy, precision, recall. From Table 3a, the experimental results show that the accuracy of COPD_AVG_FL based model outperforms the baseline CML, echoing 14.3%, 14.7% and 11.3% improvement on the three hospitals, respectively. Similarly, revealing 13.5% (14%), 14.5% (13.5%), 12% (11%) precision (recall) improvement, respectively. Experiments show that the accuracy, precision and recall of the COPD_AVG_FL based model improve by over 10% on average compared to the CML. Specially, COPD_AVG_FL based model achieves the greatest improvement on accuracy. It can be found that the developed COPD_AVG_FL system may serve as an effective tool for clinical decision-making.

**Table 3:**
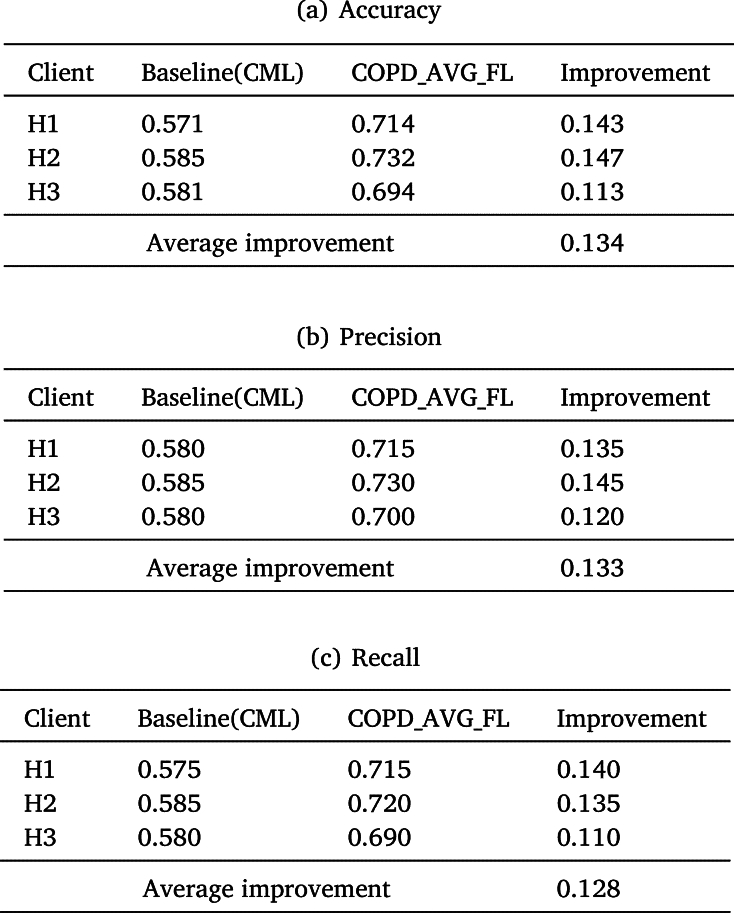
Comparsion between CML and COPD_AVG_FL on Accuracy, Precision and Recall.

**Figure 4:**
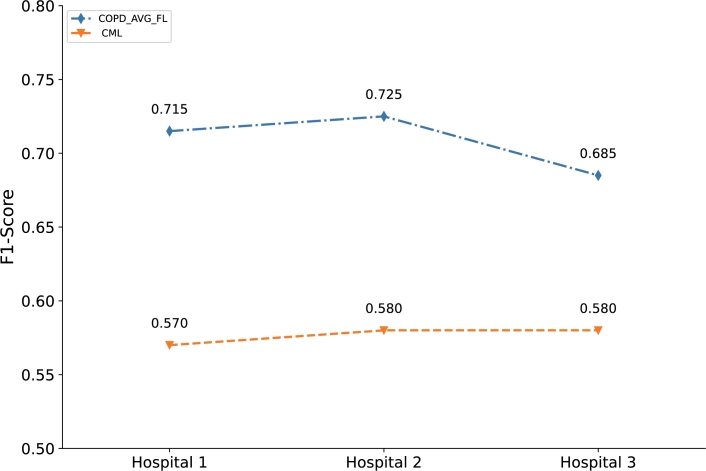
Comparison between CML and COPD_AVG_FL on F1-Score. x-axis indicates the hospitals participating in experimentation and y-axis denotes the model evaluation indicator F1-Score.

F1-Score as a common comprehensive indicator of classification model performance considers the precision and recall. The comparison of F1-Score for CML and COPD_AVG_FL is shown in Fig. 4. The F1-Score of COPD_AVG_FL based model for the three hospitals achieve 71.5%, 72.5% and 68.5%. Compared with baseline (CML), the COPD_AVG_FL based model improves the F1-Score by 14.5%, 14.5% and 10.5% on the three hospitals, respectively. According to the experimental results, the F1-Score from the COPD_AVG_FL based model outstrips the baseline (CML), which indicates that COPD_AVG_FL is effective in WITMED of COPD.

**Figure 5:**
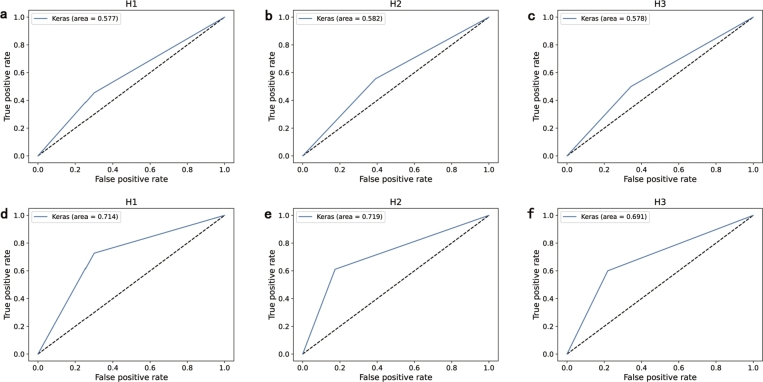
Comparsion between CML and COPD_AVG_FL on ROC. **a, b** and **c** denotes the evaluations of CML by ROC. **d, e** and **f** represents the experimental results of COPD_AVG_FL based model by ROC. The x-axis indicates the false positive rate. The y-axis denotes the true positive rate.

**Figure 6:**
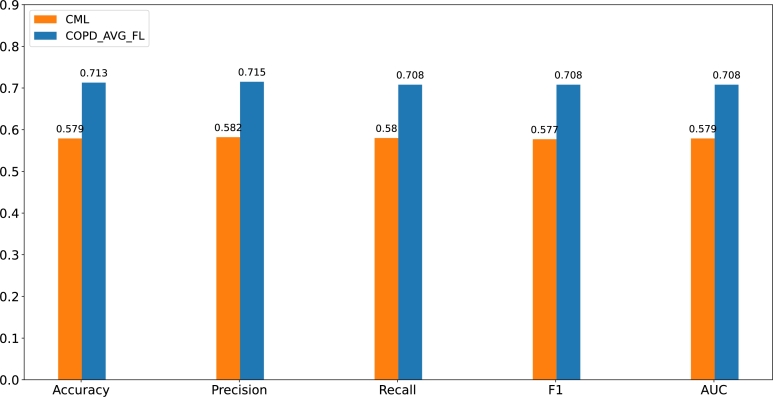
Comprehensive comparison of various indicators. The x-axis represents the various indicators. The y-axis denotes the values from various indicators.

ROC as a common binary classifier evaluation tool depicts the comparison between two operating characteristics (true positive rate and false positive rate). The comparison between CML and COPD_AVG_FL on ROC is illustrated in Fig. 5. COPD_AVG_FL based model yields the AUC of 0.714, 0.719, 0.691, respectively. Compared with CML, COPD_AVG_FL achieves a higher AUC. It is clear that COPD_AVG_FL gains the 0.137 improvement on hospital 1 from Figs. 5a, 5d. Similarly, 0.137 improvement on hospital 2 from Figs. 5b, 5e and 0.113 improvement on hospital 3 from Figs. 5c, 5f. A comprehensive comparison of the various indicators is shown in Fig. 6. On the accuracy, precision, recall, F1-Score (F1), and AUC, the proposed COPD_AVG_FL reaches an average improvement of 13.4%, 13.3%, 12.8%, 13.1%, and 12.9%, respectively. The experimental results further demonstrate the effectiveness of our proposed method.

**Figure 7:**
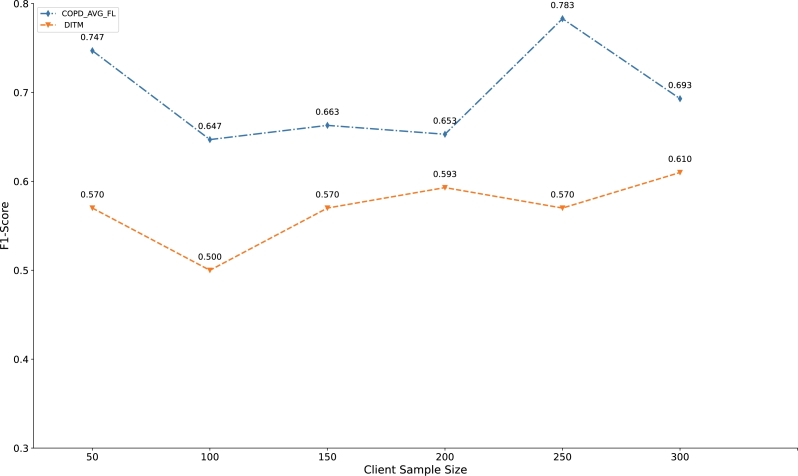
Comparison of F1-Score for CML and COPD_AVG_FL with different sample sizes. The x-axis is the sample size and y-axis denotes the model evaluation indicator F1-Score (communication rounds=350).

**Figure 8:**
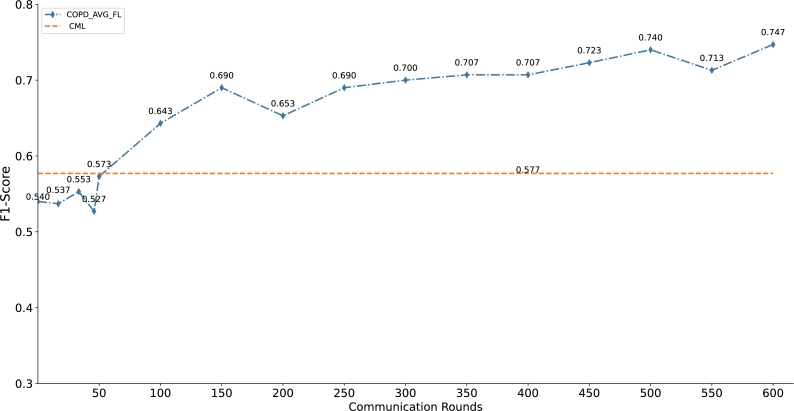
Comparison of F1-Score for CML and COPD_AVG_FL with different communication rounds. The x-axis is the communication rounds and y-axis depicts the model evaluation indicator F1-Score.

In order to evaluate the effectiveness of the proposed method, we analyze the hyperparameters (sample size, communication round and train_test_split) that may affect the performance of the architecture. To certify the gains that are presented above is robust, the comparison of F1-Score for CML and COPD_AVG_FL with different sample sizes is conducted (Fig. 7). The sample size is the independent variable and the F1-Score is the dependent variable. Fig. 7 shows that the performance of COPD_AVG_FL is superior to baseline CML with the value of sample size from 50 to 350, with a step size of 50. We can find that the performance improvement fluctuates with the increase of client sampling size, which may be related to the data quality of each medical unit. Notably, on the current dataset, although the performance improvement of the model is fluctuating, the method we proposed improves the accuracy of disease classification on the premise of protecting patient privacy. Similarly, the effect of communication round on the performance of the COPD_AVG_FL is also investigated. Fig. 8 indicates the comparison of F1-Score for CML and COPD_AVG_FL with different communication rounds. COPD_AVG_FL is inferior to CML as the communication rounds is less than or equal to 50. However, we can see that COPD_AVG_FL outperforms the baseline CML with larger communication round. With the increase of the number of communication rounds, the probability of obtaining a convergent FL architecture increases.

Further, to evaluate the stability of the proposed COPD_AVG_FL system, five train_test_split (50%:50%, 60%:40%, 70%:30%, 80%:20% and 90%:10%) are picked when the communication round equals to 350 (Table 4). As the train_test_split is set to 50%:50%, the baseline CML reaches 0.637, 0.640, 0.633 and 0.633 on accuracy, precision, recall, and F1-Score, respectively. Similarly, COPD_AVG_FL is 0.680, 0.687, 0.677, and 0.670, respectively. We find that the COPD_AVG_FL obtains the best auxiliary diagnosis performance as the train_test_split is set to 70%:30%.

**Table 4:** Comprehensive evaluation of CML and COPD_AVG_FL with comparing the train_test_split (communication rounds=350).

Train_test_split		Performance			
		Accuracy	Precision	Recall	F1-Score
50%:50%	CML	0.637	0.640	0.633	0.633
	COPD_AVG_FL	0.680	0.687	0.677	0.670

60%:40%	CML	0.626	0.627	0.627	0.623
	COPD_AVG_FL	0.660	0.657	0.657	0.657

70%:30%	CML	0.579	0.582	0.580	0.577
	COPD_AVG_FL	0.713	0.713	0.707	0.707

80%:20%	CML	0.585	0.560	0.560	0.557
	COPD_AVG_FL	0.636	0.683	0.670	0.627

90%:10%	CML	0.516	0.497	0.527	0.490
	COPD_AVG_FL	0.651	0.610	0.607	0.603

To verify whether COPD_AVG_FL exhibits heterogeneous distribution, we additionally report 95% confidence intervals for accuracy, precision, recall and F1-Score for 100 randomized tests in Table 5. The evaluation of heterogeneous distribution shows that the developed COPD_AVG_FL system is effective to build a high quality model under the premise of protecting patient privacy compared with CML.

**Table 5:** Heterogeneous distribution evaluation of COPD_AVG_FL with 100 randomized tests.

	Accuracy	Precision	Recall	F1-Score
CML	(0.606, 0.625)	(0.605, 0.624)	(0.607, 0.626)	(0.600, 0.619)
COPD_AVG_FL	(0.687, 0.706)	(0.711, 0.730)	(0.680, 0.697)	(0.666, 0.686)

## 4 Discussion

In this paper, we develop the COPD_AVG_FL system to break the data island among isomeric medical treatment combination while offering many practical privacy benefits, and build a more reliable AI model to diagnose COPD. Our experiment suggests that the proposed system contributes to the landing of WITMED in auxiliary diagnosis of COPD. However, there are several issues that need to be further discussed.

Compared with infectious disease, there is no specialized hospital for the treatment of COPD. As the characteristics of high mortality and morbidity, a large amount of COPD data is scattered and stored separately in hospitals that vary in size and level. Taking the literature review Table 6 as an example, different studies employ different features and methods, the commonality of these studies is the use of CML. The direct use of scattered data distributed in a single medical institution for data analysis and modeling may bring the problem of statistical deviationhas. Moreover, patient privacy is a major issue. A number of laws have been enacted to provide the patient privacy protection, but it also restricted the share of the COPD data among various hospitals. Furthermore, due to the high heterogeneity of COPD, it is necessary to aggregate the COPD data from different hospitals to build a robust model. Therefore, it is need to develop a distributed learning system to solve the problems above.

**Table 6:** Related works.

Author	Method	Features	Evaluation	DML
Kronborg et al.	Two-layer probabilistic model	Oximetry, etc.	average AUC of two-layer model improves by 0.11 than single-layer model.	No
Mahmudah et al.	Machine learning and regression algorithms	Microarray data	80% accuracy, 90% AUC score.	No
Wang et al.	Five machine learning models	Time span, etc.	0.80 sensitivity, 0.83 specificity.	No
Srivastava et al.	CNN-based deep learning	Mel-Frequency Cepstral Coefficients, etc.	93%. accuracy	No
Huang et al.	K-means-based Multiple LWLR model	Sulfur dioxide, etc.	9.03% prediction error.	No
Hussain et al.	Voting ensemble classifier	Sex, etc.	91.08% accuracy, 96.87% AUC score	No
Ali et al.	Soft voting score	Gender,etc.	0.71 accuracy, 0.66 F1-Score	No
Siddiqui et al.	Machine learning model,LSTM and CNN deep learning model	Age, etc.	93% accuracy.	No
Ours	Federated learning	Respiratory rate, etc.	13.4%,13.1% absolute improvement rates on accuracy, F1-Score.	Yes

Though multifarious distributed learning framework have been selected for modeling, for example, “MLlib: Fast Training of GLMs Using Spark MLlib” by Zhang, et al. [43], the AVG_FL framework is chosen in the paper on account of its simplicity and effectiveness. The developed COPD_AVG_FL system achieves good diagnosis performance under the premise of modeling with FCN network which is one of the most common neural network. Our experimental results show that the absolute improvement rates of COPD_AVG_ FL over a single data source are 13.4% (precision), 13.3% (precision), 12.8% (recall), 13.1% (F1-Score) and 12.9% (AUC), respectively. Unlike the review work, see Table 6. We focus on DML using multi-data source FL under the premise of privacy protection.

The data is collected from a single third-grade class-A hospital. Due to the limitations of financial, material and human resources, we cannot invite multiple medical units as participants (clients) in our distributed learning architecture. Therefore, how to objectively simulate the data of one medical unit into multiple medical units is another challenge in front of us. As a classical method to overcome statistical bias, random sampling technology is often used for data processing. In the study, we apply the random sampling technology to simulate the multiple medical units. In the future, we will collect more clinical data from different units to improve our COPD_AVG_FL system. Noted that being dominated by big data owner tends to cause data monopoly and makes it difficult to share data equitably with small data owner. In addition, small and medium-sized medical units are more concerned about income and patient volume, and it is not always easy to mobilize the enthusiasm of different medical units to participate FL. Therefore, how to impel small and medium-sized hospitals to join the FL mechanism requires the support of policy, government and healthcare public interest organizations.

To build an auxiliary model that can predict the deterioration of AECOPD, we collect the mild group of AECOPD and the severe group of AECOPD. The mild group of AECOPD contains 220 patients, while the severe group includes 188 patients. Using samples with a ratio close to 1:1 for modeling conforms to the data sampling criteria of statistics and machine learning, ensuring that the trained model has a small prediction bias on the current task. However, COPD is a largely heterogeneous condition, consisting of a number of pathological processes whose effects are modified by varied host susceptibility, males may be predisposed to an emphysema phenotype and females may be predisposed to an airway phenotype of COPD. In this study, the male-to-female population imbalance may lead to modeling bias. The correlation between gender and specific COPD diseases is a promising research topic, and in the future, we will collect male-to-female population balance data to explore this correlation.

COPD is a progressively developing condition, and studying it in stages indeed offers advantages for a more comprehensive analysis of this disease. Collecting staged COPD data requires regular patient follow-ups and long-term tracking throughout the entire process, which incurs additional human and time costs. Staging provides valuable prognostic information, helping clinicians predict disease progression and the likelihood of exacerbations. In our research, our focus is on the premise of protecting patient privacy, integrating scattered patient data from different medical units for high-quality modeling. In future research endeavors, with the support of government funding and increased financial investment, we intend to collect the stage COPD data to achieve real-time monitoring of COPD conditions.

Given the characteristics of distributed learning, network traffic load is needed to transmit the parameter to achieve the convergent COPD_AVG_FL architecture. Therefore, communication network traffic (network bandwidth) is a necessary facility for COPD_AVG_FL training. Generally, the larger the scale of the COPD_AVG_FL is, the more traffic it consumes. If the network bandwidth cannot support the framework communication, the package lose will cause the failure of parameter update, resulting in a series of problems, for example, failure to converge the model. Fortunately, the proposed COPD_AVG_FL only requires 350 rounds of communication to achieve convergence, which will not bring a huge burden to the network of medical units. In the future, with the construction and development of communication technology (2G, 3G, 4G or 5G), the deployment of our architecture in real medical scenarios will become possible.

Our study focuses on the early prediction of diagnosis of the deterioration and death risk, which holds significant clinical importance. To achieve this goal, we primarily collect data from AECOPD patients. The experimental results demonstrate that the framework we proposed performs favorably in predicting the risk of deterioration and mortality in AECOPD. During the data collection phase of our study, we do not collect clinical data related to asthma in our real-world dataset. Therefore, our model lacks the ability to differential diagnosis with other diseases, such as asthma. Note that the focus of this paper is the utilization of FL techniques to achieve the early prediction of diagnosis of the deterioration and death risk. On the premise of protecting patient privacy, we integrate scattered patient data from different medical units for high-quality modeling. In the future, we will collect data related to asthma and asthma-COPD overlap syndrome. By collecting this data, we can retrain our framework, the system can potentially be expanded to assist in diagnosing other conditions, such as asthma and hypertension, and possibly differentiate between asthma and asthma-COPD overlap syndrome.

## 5 Conclusion

In the work, we present COPD_AVG_FL, a distributed system using FedAvg to achieve high-quality models to assist the diagnosis of COPD. The experiments show that the models trained by the COPD_AVG_FL outperforms the CML models in a few communication rounds. It must be emphasized that the quantity and quality of training data are the key factors affecting the performance of the COPD_AVG_FL system. The auxiliary diagnosis performance of COPD_AVG_FL can be improved by collecting high-quality data [44]. Future work is to build a more robust model by introducing the weight of data quantity, data quality, or the combination of both.

## Funding

Scientific research platforms and projects of colleges and universities in Guangdong Province (No. 2021ZDZX3016). Higher education special (No. 2022GXJK287). Featured innovation projects (No. 2023KTSCX098).

## Ethics approval

The work received approval and informed consent waiver from the Ethics Committee of The Third Affiliated Hospital, Sun Yat-sen University (TAHSYU) Institutional Review Board (IRB), protocol [2019]-02-334-01, which waived informed consent owing to the retrospective cohort study design. All patients did not provide written signed informed consent. All methods were carried out in accordance with relevant guidelines and regulations.

## CRediT authorship contribution statement

**Junfeng Peng:** Methodology, Conceptualization. **Xujiang Liu:** Writing – original draft, Methodology. **Ziwei Cai:** Writing – original draft, Methodology. **Yuanpei Huang:** Writing – original draft, Visualization. **Jiayi Lin:** Writing – original draft, Visualization, Conceptualization. **Mi Zhou:** Validation, Data curation. **Zhenpei Xiao:** Investigation, Data curation. **Huifang Lai:** Validation. **Zhihao Cao:** Validation. **Hui Peng:** Validation. **Jihong Wang:** Writing – original draft, Visualization. **Jun Xu:** Validation, Data curation.

## Declaration of Competing Interest

The authors declare that they have no known competing financial interests or personal relationships that could have appeared to influence the work reported in this paper. The work described in this paper has been supported by the Scientific research platforms and projects of colleges and universities in Guangdong Province (No. 2021ZDZX3016), Higher education special (No. 2022GXJK287), and Featured innovation projects (No. 2023KTSCX098).

## Data Availability

Data will be made available on request.
